# Case report: Severe presentation of a syndromic congenital bilateral upper eyelids eversion

**DOI:** 10.1016/j.amsu.2022.103279

**Published:** 2022-01-25

**Authors:** Jihene Sayadi, Ines Malek, Yosra Abid, Dhouha Gouider, Manel Mekni, Amel Chebbi, Leila Nacef

**Keywords:** Upper eyelids eversion, Congenital eyelids abnormality, Congenital ectropion, Umbilical hernia, Clubfoot, Case report

## Abstract

**Introduction:**

and importance: Congenital upper eyelid eversion (CUEE) is a rare congenital condition characterized by everted upper eyelids with prominent chemosis. The authors present the first case of concurrent upper eyelids eversion, umbilical hernia, and clubfeet.

**Case presentation:**

A four-hour-old newborn male presented with bilateral red upper eyelids swelling. Ophthalmic examination revealed bilateral upper eyelids eversion and severe bilateral chemosis. The further pediatric evaluation showed a painless reducible umbilical hernia and clubfeet. Treatment of the eyelids eversion was conservative, combining topical steroids, antibiotics and lubricants. Chemosis reduced progressively. We obtained a complete resolution on day 21. We referred the neonate to the pediatric surgery, and orthopedic department for umbilical hernia and clubfeet management.

**Clinical discussion:**

Most infants with CUEE may show excellent anatomic and functional results with conservative treatment if managed timely and promptly.

**Conclusion:**

The innocuous appearance of CUEE must not prevent clinicians from investigating possible systemic associations and initiating appropriate treatment.

## Highlights

• Congenital upper eyelid eversion is a very rare congenital condition, few cases have been published in the literature.
• This condition is usually isolated. However, a higher prevalence is reported in Down syndrome and colloidal skin disease.
• To the best of our knowledge, we present the first case of concurrent upper eyelids eversion, umbilical hernia and clubfeet.
• Management is often conservative, most infants with congenital upper eyelid eversion may show excellent results if managed timely and promptly.

## 1 Introduction

Congenital upper eyelid eversion is an uncommon condition characterized by a complete outward turning of the upper eyelid, exposing the palpebral conjunctiva. It is associated with severe conjunctival chemosis [1]. This congenital abnormality is usually isolated. However, the authors reported a higher prevalence in newborns with Down syndrome and colloidal skin disease. Moreover, black infants would be the most affected [2,3].

Our case report aimed to describe a previously unreported association of CUEE, umbilical hernia, and clubfeet in a white neonate. It also highlighted the potentially severe ocular impact of CUEE and its therapeutic options, including conservative management.

This case report has been reported in line with the SCARE 2020 criteria [4].

## 2 Presentation of case

A four-hour-old white male neonate with no family history was referred to our emergency department for severe red swelling in his upper eyelids, precluding eyeballs identification. He was the third child of a 26-year-old woman. Pregnancy was uneventful and regularly followed up. The child was full-term. He was born through vaginal delivery with no instrumentation.

**Fig. 1:**
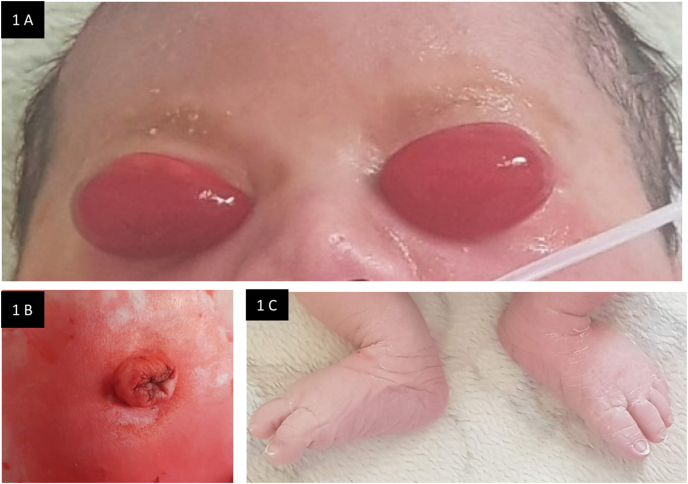
A. Clinical photograph at presentation showing severe bilateral chemosis with complete eversion of the upper eyelids. B. Reducible umbilical hernia. C. Bilateral excessive dorsal inflection consistent with clubfeet.

Ophthalmic examination revealed severe bilateral chemosis with complete eversion of the upper lids and exposure of the tarsal plate (Fig. 1A). The eyelids were then carefully parted to check the eyeballs. Both globes were normal-sized. Anterior segment evaluation was unremarkable in both eyes.

Further detailed pediatric assessment disclosed a painless reducible umbilical hernia (Fig. 1B). Both feet examination showed excessive dorsal inflection consistent with clubfeet (Fig. 1C).

We diagnosed an unknown association of CUEE, umbilical hernia, and clubfeet. A genetic study could not be performed due to socio-economic issues. As we timely established the diagnosis at birth, the prognosis was good. There was no other diagnosis to discuss.

Management of the eyelids eversion was conservative. The patient was started on moist dressings, a topical antibiotic-corticosteroid ophthalmic ointment, and lubricants three times per day. The medical treatment aimed to reduce chemosis, prevent infection, and lubricate the cornea.

**Fig. 2:**
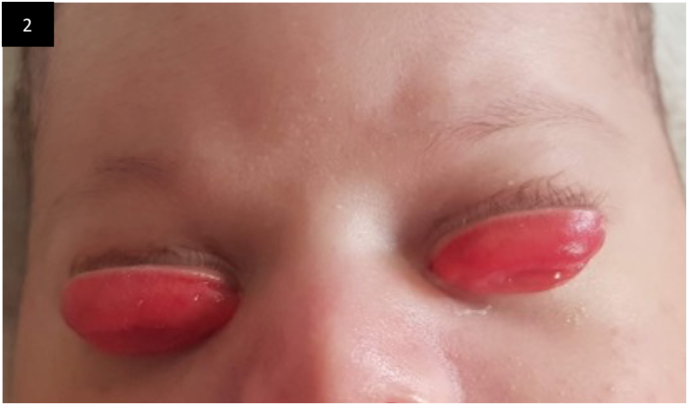
Clinical photograph on the seventh day from the presentation showing partial resolution of the chemosis.

Chemosis reduced progressively within seven days allowing manual repositioning of the eyelids (Fig. 2). Complete resolution was achieved on both eyes at day 21 without ocular sequelae.

We referred the neonate to the pediatric surgery and orthopedic department for associated abnormalities management.

The parents were relieved after the fast resolution of the swelling as they thought it was a potentially blinding disease. They will continue the treatment and clinical follow-up in the ophthalmology and surgery departments.

## 3 Discussion

Congenital upper eyelid eversion is an uncommon condition, first described by Adams in 1896 as “double congenital ectropion” [1]. It is typically bilateral and present at birth. Nevertheless, late presentations and some unilateral cases have been described [2,5].

The exact pathogenesis of CUEE is not yet known. Anatomical variations of the upper eyelid are mainly involved. They include hypotonia of the orbicularis oculi, absence of effective lateral canthal ligaments, lateral elongation of the eyelid, and vertical shortening of the anterior lamella or lengthening of the posterior lamella. The failure of the orbital septum to fuse with the levator aponeurosis can also be responsible for CUEE [3,5]. However, a 9-day-neonate with CUEE detailed post-mortem examination failed to identify any of these anatomic lid abnormalities. It led the authors to discuss other hypotheses including prolonged labor and birth trauma [3,5,6].

Once everted, vascular congestion within the eyelid causes chemosis and swells the lid tissues enough to exacerbate orbicularis spasm. It leads to a vicious circle of worsening lid eversion. Thus, the eyelid cannot be repositioned [3,5,6].

To the best of our knowledge, no one reported the association of CUEE, umbilical hernia, and clubfeet. Authors described clubfeet in several congenital syndromes such as Down syndrome, probably due to ligamentous laxity [7]. However, congenital umbilical hernias are common in childhood. It can be an incidental finding in the current case [8].

Although its etiology remains unclear, clinicians should be aware of this unreported congenital abnormality of the ocular, abdominal and musculoskeletal system.

Congenital upper eyelid eversion is usually benign. Nevertheless, it can expose to keratopathy, corneal scarring, and perforation leading to severe ocular morbidity [9].

The conservative management often aims to prevent infection and reduce sufficiently conjunctival chemosis. It usually allows spontaneous upper eyelids inversion [3,5,10].

The different treatment modalities are moist dressings, pressure patching, topical antibiotics, and lubricant. Antibiotic is an adjunct treatment that prevents exposed ocular surface infection. Some authors also proposed topical 5% hypertonic NaCl to dehydrate the swollen conjunctiva [3,10].

In the current case, topical corticosteroids helped address any inflammatory component of the subconjunctival edema. Witherspoon et al. recommended a subconjunctival injection of steroids, lidocaine, and epinephrine for resistant conjunctival chemosis [10]. Daniel et al. were the first to use systemic steroids in CUEE. They speculated that the systemic route was less risky than local injection in neonates [3].

We often reserve surgical treatment for refractory cases to a conservative approach. It includes scarification of the exposed conjunctivae, temporary tarsorrhaphy, fornix sutures, full-thickness skin graft to the upper lid, and subconjunctival hyaluronic acid injection [2,9,10].

To conclude, this case report is quite interesting, as we describe a new congenital syndromic association. Management of CUEE is still controversial, mainly due to its scarcity.

## 4 Conclusion

Most infants with CUEE usually show excellent anatomic and functional results. We can avoid risky and unnecessary surgery with early and prompt management. Moreover, the innocuous appearance of CUEE must not prevent clinicians from investigating possible systemic associations.

## Ethical approval

This case report followed the ethical standards of the declaration of Helsinki and its later amendments. Anonymous case reports are approved by the ethics committee of our institution as long as we have written consent.

## Sources of funding

Not applicable.

## Author contributions

Patient's management: JS and AC.

Data collecting: MM and JS.

Manuscript drafting: YA, JS, DG, and IM.

Manuscript revision: JS, MM, DG, IM, and LN.

All authors approved the final version of the manuscript.

## Trial registry number

Name of the registry:
Unique Identifying number or registration ID:
Hyperlink to your specific registration (must be publicly accessible and will be checked):

## Provenance and peer review

Not commissioned, externally peer-reviewed.

## Consent

We obtained written informed consent from the patient's father to publish this case report and accompanying images. A copy of the written is available for review by the Editor-in-Chief of this journal on request.

## Guarantor

Dr. Jihene Sayadi is the corresponding author and the guarantor of this case report.

## Declaration of competing interest

No conflict of interest.
